# A school-curriculum-based exercise intervention program for two years in pre-pubertal girls does not influence hip structure

**DOI:** 10.1186/1476-5918-7-8

**Published:** 2008-04-28

**Authors:** Gayani Alwis, Christian Linden, Susanna Stenevi-Lundgren, Henrik G Ahlborg, Magnus Dencker, Jack Besjakov, Per Gardsell, Magnus K Karlsson

**Affiliations:** 1 Clinical and Molecular Osteoporosis Research Unit, Department of Clinical Sciences, Lund University, Sweden; 2 Department of Clinical Physiology, Malmö University Hospital, SE-205 02 Malmö, Sweden; 3 Department of Radiology, Malmö University Hospital, SE-205 02 Malmö, Sweden

## Abstract

**Background:**

It is known that physical activity during growth has a positive influence on bone mineral accrual, and is thus possibly one strategy to prevent osteoporosis. However, as bone geometry, independent of areal bone mineral density (aBMD), influences fracture risk, this study aimed to evaluate whether hip structure in pre-pubertal girls is also affected by a two-year exercise intervention program.

**Methods:**

Forty-two girls aged 7–9 years in a school-curriculum-based exercise intervention program comprising 40 minutes of general physical activity per school day (200 minutes per week) were compared with 43 age-matched girls who participated in the general Swedish physical education curriculum comprising a mean of 60 minutes per week. The hip was scanned by dual energy X-ray absorptiometry (DXA) and the hip structural analysis (HSA) software was applied to evaluate bone mineral content (BMC, g), areal bone mineral density (aBMD, g/cm^2^), periosteal diameter, cross-sectional area (CSA, cm^2^), section modulus (Z, cm^3^) and cross-sectional moment of inertia (CSMI, cm^4^) of the femoral neck (FN). Annual changes were compared. Subjective duration of physical activity was estimated by questionnaire and objective level of everyday physical activity at follow-up by means of accelerometers worn for four consecutive days. All children remained at Tanner stage 1 throughout the study. Group comparisons were made by independent student's *t*-test between means and analyses of covariance (ANCOVA).

**Results:**

At baseline, the two groups did not differ with regard to age, anthropometrics or bone parameters. No between-group differences were observed for annual changes in the FN variables measured.

**Conclusion:**

A two-year school-based moderately intense general exercise program for 7–9-year-old pre-pubertal girls does not influence structural changes in the FN.

## Background

Physical activity during growth induces benefits in bone mineral accrual and bone structure [1-4]. This is of clinical relevance as both traits are important for bone strength [5]. However, measurements of bone mineral content (BMC) and bone size by dual energy X- ray absorptiometry (DXA) must be interpreted with care, as there is a relationship between the traits so that a bigger bone is related to a greater BMC. Furthermore, the growing skeleton responds more favorably to mechanical loading than the adult skeleton [6] and the pre- and early pubertal years seem to be the period with the greatest responsiveness [1,7,8]. Most exercise trials, however, have focused on the accrual of bone mineral [9-12], a study design that may underestimate the effects of training, as bone mineral does not take bone structure into account. Bone structure at the femoral neck (FN) can be evaluated by the DXA-based hip structural analysis (HSA). There are few previous exercise intervention studies in children that have used this technique, some supporting [13,14], others refuting [15,16] the influence of exercise during growth on the bone structure at the FN, and all with shorter follow-up than the current study. The reports published so far from the Pediatric Osteoporosis Prevention (POP) study in girls [11,12] have only used the standard dual energy X-ray absorptiometry (DXA) software. These publications have reported an exercise-induced beneficial outcome in bone mineral accrual and a greater gain in skeletal width of the third lumbar vertebra (L3), with a trend toward a beneficial effect on FN width.

However, the POP study has so far not reported the effect of physical activity on the FN structure. By introducing the HSA software [17-19] to specifically evaluate the FN structure, we were better able to estimate the biomechanical properties of the FN than if only measuring FN width. Estimation of biomechanical calculation is of great interest, as an exercise induced benefit in FN width reflects a much greater benefit in cross-sectional moment of inertia (CSMI). CSMI is an estimate of the ability of the FN to withstand bending forces, proportional to the fourth power of the radius [5,20].

Thus, the aim of this trial was to ascertain whether a moderately intense exercise intervention program for two years in pre-pubertal girls [12] could also influence the biomechanical indices of the FN. Secondly, because of the well-known difficulty in estimating physical activity in young children [21,22], accelerometer data were included to quantify the amount of moderate or vigorous activity. We hypothesized that a moderately intense exercise intervention program would be beneficial for FN structure and that there ought to be more both moderate and vigorous activity in the intervention group.

## Methods

The Malmö Pediatric Osteoporosis Prevention (POP) study is a prospective controlled school physical education intervention study that follows skeletal traits in a population-based cohort of pre-pubertal children. The study design has previously been thoroughly reported with discussion of the accrual of lumbar spine and FN bone mineral and gain in bone width [11,12]. All four invited neighboring schools included were government-funded, with the children allocated to the school according to their residential address and with the Swedish standard curriculum for school physical education classes before study start. None of the invited schools denied participation. One of the schools was then invited to participate as the intervention school. This school instantly accepted, even if they had to modify their curriculum by increasing the amount of physical educational classes. In other words, we did not choose a school with an already high level of physical activity as the intervention. The cohort could thus be regarded as a cluster of convenience, the schools being the clusters and the convenience being that they all came from the same neighborhood.

In the intervention school, all girls in grades one and two were included in a school-curriculum-based exercise intervention program. Of 61 girls, 55 agreed to participate in the baseline measurements. One girl was excluded, as she was eleven months younger than the second youngest girl. After two years, five girls declined participation, leaving 49 girls with a mean age of 7.6 ± 0.6 years (range 6.5–8.7) at baseline for this report. The volunteers from the control schools included 64 girls within the same grades. After two years, 13 of these girls had moved or declined further participation, and one was excluded as she was being treated with a medication known to affect bone metabolism. Thus, 50 girls with a mean age of 7.9 ± 0.6 (range 6.8–8.9) at baseline were included as controls. Due to the technical problems when DXA scanning of small children caused by inconsistency in limb positioning and location of the region of interest [23], we advocated the method introduced by Beck et al. when evaluating FN width [18]. This method excludes biologically unlikely values, 3 standard deviations (SD) above or below the mean. This resulted in exclusion of four scans of the hip in the intervention group and six in the control group. In addition, missing scans or scans with obvious technical errors were found in three hip scans in the intervention group and one in the control group. As a result, 42 cases and 43 controls are included in this report.

All girls were Caucasians without any disease or medication known to influence bone metabolism. All girls stayed at Tanner stage 1 during the study, according to the assessment of pubertal stages done by our research nurses [24]. As described in previous publications [11,12], there were no differences regarding height, weight and body mass index (BMI) when a drop-out analysis compared the study participants and the non-participants and the children in the intervention group and the control group.

The Swedish school physical educational curriculum includes a variety of activities such as ball games, running, jumping and climbing. The school physical education intervention included these same types of activities, which were performed under the supervision of the class teacher but the normal mean curriculum time of 60 minutes per week was increased to 40 minutes per day (200 minutes per week). In the control schools, the same activities were used, although limited to only one or two sessions (60 minutes) per week (Table 1), the mean level of the standard Swedish school curriculum.

**Table 1:** Data as regard the level of physical activity at baseline and follow-up.

	**Intervention**	**Control**	***p*-value**
**Baseline data**			

Numbers	42	43	
Age	7.7 (0.6)	7.9 (0.6)	0.08
**Organized physical activity evaluated through questionnaire (hrs/w)**			
School curriculum	3.3	1.0	**< 0.001**
Outside the school	0.7 (0.6)	1.4 (1.7)	**0.009**
Total physical activity	4.0 (0.6)	2.4 (1.7)	**< 0.001**
			
**Follow-up data**			

Numbers	42	43	
Age	9.7 (0.6)	9.9 (0.6)	0.14
**Organized physical activity evaluated through questionnaire (hrs/w)**			
School curriculum	3.3	1.0	**< 0.001**
Outside the school	1.4 (1.4)	2.0 (2.1)	0.13
Total physical activity	4.7 (1.4)	3.0 (2.1)	**< 0.001**
			
**Accelerometer data**			

Numbers	41	40	
Recording time per day (hours/day)	11.8 (1.4)	12.0 (1.3)	0.50
Mean activity/day (counts per minutes)	644 (192)	590 (121)	0.13
Moderate to vigorous activity (> 3 METs; min/day)	193 (47)	185 (37)	0.38
Vigorous activity (> 6 METs; min/day)	34 (15)	35 (13)	0.77
> 5000 counts per minutes/day	17.9 (9.9)	16.8 (8.5)	0.58
> 6000 counts per minutes/day	12.5 (7.6)	10.5 (6.2)	0.19
> 10,000 counts per minutes/day	2.6 (2.5)	1.0 (1.2)	**< 0.001**

Data presented as mean (SD).

The methodology of physical activity measurement has previously been presented in detail [25-27]. Physical activity was assessed using the MTI (Manufacturing Technology Incorporated, Fort Walton Beach, FL, USA) accelerometer, model 7164. The children were instructed to wear the accelerometers for four consecutive days, including both weekdays and weekends. Accelerometers were not used during some activities such as swimming. The children were also given specific instructions to put them on as soon as they got out of bed in the morning and take them off when they went to bed at night. Accelerometers were programmed to start recording in the morning of the first day and measure continuously for 4 days. However, there are weaknesses in the registration of activity. The accelerometers were not used during sleep or when performing any activity in water. Similar activity could thus be recorded differently. Trampoline jumping into a pool would not be registered whereas trampoline training on ground would. Around of 70% of the recorded accelerometer time included school days (also including the school physical educational classes) and 30% included weekend days. Furthermore, the data collection was performed a mean 8 days apart from the follow-up BMD measurements. One girl in the intervention and three girls in the control group did not complete four whole days so we had to exclude them from the study. Accelerometer data is then averaged over a period called an epoch. A recording epoch of ten seconds was selected for this study. SAS-based software (SAS Institute Inc, Cary, NC, USA) was used to analyze all accelerometer data. This software automatically deletes missing data, defined as continuous sequences of 60 consecutive epochs (i.e. 10 minutes) or more with zero counts. This was done based on the assumption that all such sequences of zeros lasting longer than ten minutes were caused by the accelerometer not being worn. In order to minimize inter-instrumental variation, all accelerometers were calibrated against a standardized vertical movement. Mean activity was considered to be the total accelerometer counts per valid minute of monitoring (mean counts/min; cpm). Age- and body-mass-specific cut-off points exist for accelerometer counts representing activity of varying intensities, and these cut-off points made it possible to roughly estimate the number of minutes the child was engaged in activity above a specific intensity threshold [28,29]. Time spent performing above 3 Metabolic Equivalents (METs) was considered to reflect moderate-to-vigorous activity (MVPA), and time spent above 6 METs was considered to reflect vigorous activity (VPA). Cut-off points used for all children were > 167 counts/epoch for MVPA and > 583 counts/epoch for VPA [28,29].

The baseline measurements in the intervention school were made before the intervention was initiated and the follow-up measurements two years later. The three neighboring control schools were evaluated in the same way. During the dual X-ray absorptiometry (DXA, DPX-L version 1.3z, Lunar^®^, Madison, WI) measurements the children were dressed in light clothes and no shoes. Pediatric software was used for children with a weight below 35 kg. Total lean mass and total fat mass were estimated from the DXA total body scan. All standard image files of the proximal femur were analyzed by one technician, using the hip strength analysis (HSA) software, provided by Lunar Instruments Corporation (Madison, WI), a technique described in detail in the previous publications [16]. In this trial we report the bone mineral content (BMC, g) and areal bone mineral density (aBMD, g/cm^2^), width (mm), cross-sectional area (CSA, cm^2^), section modulus (Z, cm^3^) and cross-sectional moment of inertia (CSMI, cm^4^) of the femoral neck (FN).

Daily calibration of the DXA machine was performed with the Lunar^® ^phantom. The research technicians in our research group performed all the measurements and the software analyses. The coefficients of variation for the FN traits, evaluated by duplicate measurements in 13 healthy children with a mean age of 10 years, were as follows: BMC 1.4%, aBMD 1.6%, periosteal diameter 1.5%, CSA 2.2%, Z 6.2%, CSMI 6.2%, and for total body fat mass 3.7% and total body lean mass 1.5%.

Body weight was measured with an electric scale to the nearest 0.1 kg and body height by a wall-tapered height meter to the nearest 0.5 cm. The previously advocated lifestyle questionnaire was also used in this report [11,12]. Questionnaires were given at baseline and at follow-up. Duration of physical activity just after the intervention was initiated was specifically registered in the baseline questionnaire. The duration of physical activity at follow-up was specifically registered in the follow-up questionnaire. The total time spent in physical activity was calculated as weekly time spent in training within the school curriculum and weekly time spent in organized sports activities outside of school. The children answered the questionnaire together with their parents, in order to minimize recall error.

Informed written consent was obtained from parents or guardians of participants prior to the study start. The study was approved by the Ethics Committee of Lund University (LU 453-98; 1998-09-15), Sweden, and conducted according to the Helsinki Declaration of 2000. The Swedish Data Inspection Board approved both the data collection and the database.

Statistical calculations were performed with Statistica^®^, version 6.1 (StatWin^®^). Data are presented as mean ± SD. Baseline data, follow-up data and changes per 365 days were compared between the cases and the controls by student's *t*-test. Baseline data were adjusted for chronological age and organized physical leisure time activity at baseline with analyses of covariance (ANCOVA). Follow-up data and annual changes were adjusted for chronological age and organized physical leisure time activity at baseline and increments in height and weight in the follow-up evaluations by an ANCOVA. A p-value of < 0.05 was regarded as a statistically significant difference.

## Results

As shown in Table 1, at baseline and at follow-up, total duration of physical activity estimated through questionnaire was higher in the intervention group than in the control group. In contrast, at follow-up, there was no difference in total amount of daily activity measured by accelerometers, while the intervention group was presented with a higher proportion of the most intense activities, above 10,000 cpm.

As shown in Table 2, there were no differences at baseline in anthropometrics or bone parameters between the intervention and control groups. The results remained after adjustment for age and organized leisure time activity at baseline (data not shown). Also, there were no significant differences in the annual changes of the FN skeletal traits when the intervention and the control groups were compared. The results remained after adjustment for age and organized leisure time activity at baseline and changes in weight and height during the study period (data not shown).

**Table 2:** Anthropometry, bone mineral parameters and biomechanical calculations of femoral neck.

	**Intervention**	**control**	***p*-value**
**Baseline**	(N = 42)	(N = 43)	

**Total body**			
Weight (kg)	27.8 (5.4)	26.5 (4.3)	0.22
Height (cm)	128.8 (5.1)	128.7 (7.5)	0.94
Lean mass (kg)	20.1 (2.3)	20.0 (2.6)	0.90
Fat mass (kg)	5.3 (3.7)	4.5 (2.4)	0.28
**Femoral neck**			
BMC (g)	2.524 (0.468)	2.536 (0.497)	0.91
BMD (g/cm^2^)	0.722 (0.091)	0.701 (0.090)	0.30
Cross-sectional area (CSA, cm^2^)	0.909 (0.141)	0.889 (0.152)	0.54
Periosteal width (cm)	2.424 (0.162)	2.463 (0.225)	0.37
Section modulus (Z, cm^3^)	0.279 (0.064)	0.272 (0.082)	0.66
Cross-sectional moment of inertia (CSMI, cm^4^)	0.341 (0.096)	0.338 (0.130)	0.90
			
**Follow-Up**	(N = 42)	(N = 43)	

**Total body**			
Weight (kg)	35.6 (8.0)	32.5 (5.7)	**0.04**
Height (cm)	141.0 (6.5)	139.7 (8.0)	0.45
Lean mass (kg)	24.6 (3.5)	23.6 (3.0)	0.18
Fat mass (kg)	8.9 (5.8)	6.3 (3.4)	**0.01**
**Femoral neck**			
BMC (g)	3.146 (0.506)	3.030 (0.518)	0.30
BMD (g/cm^2^)	0.803 (0.080)	0.789 (0.103)	0.50
Cross-sectional area (CSA, cm^2^)	1.100 (0.175)	1.067 (0.187)	0.41
Periosteal width (cm)	2.640 (0.226)	2.593 (0.190)	0.30
Section modulus (Z, cm^3^)	0.377 (0.085)	0.368 (0.099)	0.66
Cross-sectional moment of inertia (CSMI, cm^4^)	0.506 (0.154)	0.483 (0.159)	0.49
			
**Annual Changes**	(N = 42)	(N = 43)	

**Total body**			
Weight (kg)	3.9 (1.6)	3.0 (1.1)	**0.006**
Height (cm)	6.0 (1.1)	5.6 (0.9)	0.06
Lean mass (kg)	2.3 (0.8)	1.8 (0.5)	**0.003**
Fat mass (kg)	1.8 (1.3)	0.9 (0.7)	**0.001**
**Femoral neck**			
BMC (g)	0.310 (0.234)	0.252 (0.236)	0.25
BMD (g/cm^2^)	0.040 (0.033)	0.045 (0.042)	0.62
Cross-sectional area (CSA, cm^2^)	0.095 (0.065)	0.091 (0.078)	0.77
Periosteal width (cm)	0.107 (0.107)	0.067 (0.113)	0.10
Section modulus (Z, cm^3^)	0.049 (0.030)	0.049 (0.042)	0.98
Cross-sectional moment of inertia (CSMI, cm^4^)	0.082 (0.057)	0.074 (0.067)	0.54

Comparison between the intervention group (n = 42) and the control group (n = 43) in anthropometrics, bone mineral parameters and biomechanical calculations in femoral neck at baseline, at two year follow-up and in annual changes during the follow-up period. Data presented as mean ± SD.

## Discussion

The previously published paper following the girls for two years reported a significant exercise-induced beneficial effect in FN bone width when comparing unadjusted data and a trend for exercise-induced benefit when comparing data adjusted for differences in age at baseline and growth [12]. In contrast, this study refutes that there are any significant exercise-induced structural FN benefits in the intervention group. The discrepancy could be due to the outliers being excluded in this study as well as the use of different FN software in the two reports.

The different outcome in lumbar spine [12] compared to FN, as presented in this report, when following girls for 2 years in the POP study could be due to several reasons. There could have been a different mechanical load in the two regions. This could account for both ground reaction forces and muscle load. The different proportions of trabecular and cortical bone in lumbar spine and FN [30] could also be an another reason as trabecular bone is more prone to respond to mechanical load than cortical bone [2,8-11,16]. The structural FN data presented in this trial introduce important new knowledge, as previous reports have suggested that exercise-induced structural benefits may have been underestimated when only aBMD or the standard hip DXA software are used [7]. However, this study opposes that view.

The FN structural data in this report contradict some publications about growing children [13,31] but totally or partly support others [14-16]. The discrepancies in the conclusions when the trials are compared could be due to differences in the study designs. The maturational level of the girls was different in the trials, and it is known that exercise-induced skeletal benefits are most obvious in early pubertal or in pre-menarcheal girls [8,14,32,33].

The intensities and the proportions of osteogenic activities in the cited exercise programs were different, and it is known that high-magnitude repetitive interventions confer more benefits than repeated endurance training [13,14,33-35]. There are however studies that have evaluated the ground reaction forces as one attempt to quantify the level of physical activity in exercise intervention trials [10,35,36]. However, the aim of the current study was not to estimate the osteogenic effect of specific activities but to evaluate whether a general school-based exercise intervention program could be used as a population-based prevention strategy for low bone strength.

The follow-up periods in the cited studies vary between 7 and 24 months, a fact that could also influence the outcome. The structural estimate of the FN by HSA, as the HSA is based on a two-dimensional scanning technique [18,19], may also differ from evaluation by three-dimensional techniques such as quantitative computed tomography (QCT) and magnetic resonance imaging (MRI). However, these techniques predominantly estimate the peripheral skeleton, predominantly the lower leg and forearm. Apart from different techniques, it also seems as if the response to mechanical load is different in different skeletal regions [7,37]. The limited precision of the HSA software could also influence the outcome and thus influence our inferences.

The intervention program, as estimated by the questionnaire, led to a higher duration of exercise per week but not to a higher level of activity estimated by four days of accelerometer measurements (Table 1). Today there is no consensus as to how physical activity should be registered in children, as both objective measurements of physical activity and self-reported methods are associated with flaws [21,22,25,38]. However, the longer duration of physical activity suggested by the questionnaire and the previously reported bone mass benefits found in the lumbar spine [11,12] suggest that the intervention program actually resulted in a higher level of physical activity. The accelerometer data at least partly support this view when reporting a higher proportion of the most intense activities (> 10,000 cpm) in the intervention group (Table 1). However, the duration of the level with the highest intensity spans such a short period that the data must be interpreted with great care. In spite of this, the finding is of considerable interest, as it is known that a short duration of highly intense load is of greater importance than the total duration of activity when trying to improve skeletal strength [39-44].

### Study strength

The prospective population-based design, increasing our ability to draw generalized inferences, must be regarded as a strength, while the inability to perform individual randomization must be regarded as a weakness, both facts extensively discussed in our previous reports [11,12]. The similarity in anthropometric characteristics between individuals who did or did not participate in this study, and between the intervention and the control group, provides further support that our inferences could be generalized. The use of accelerometers, as an objective estimate of daily physical activity, is also positive compared to only subjective estimates of the level of physical activity [21,22].

### Study limitations

When interpreting accelerometer data, the registrations only span a few days, at the end of the study period, perhaps not reflecting the actual activity level during the two-year study period. Furthermore, some types of activities are not captured by the technique [25,38]. The higher spare time activity in the control group could also mask eventual beneficial osteogenic effects in the intervention group. However, this seems of less probability as all results remained after adjustment for differences in spare time activity at baseline. Furthermore, it would have been beneficial if we by the questionnaire had been able to define the different type of spare time activities, not only provide the duration of the organized training activities. Also, it had been advantageous if we had been able to provide accelerometer data separately as regard the activities level during the school time, during leisure time, during weekdays and during weekends. The HSA technique with its technical limitations should also be highlighted. Inconsistent positioning of the limb or the region of interest results in errors [18,19]. A small width error results in large errors in the estimates of CSMI, as CSMI is proportional to the fourth power of the radius [5,20]. This is the reason why we excluded outliers as recommended by Beck et al. [18]. The estimates of the periosteal dimension are also derived from a two-dimensional image, but the skeleton can expand in other directions [7,45], which may not be captured by the HSA. With these limitations in mind, HSA data should be regarded as one method on which to focus interest, not only on the amount of bone mineral but also FN structure.

## Conclusion

A two-year moderately intense school-based exercise intervention program in pre-pubertal girls seems not to influence the FN structure, possibly due to the fact that the level of physical activity in this cohort of children was high, independent of being in the intervention or control group. The study also highlights the difficulties in the estimation of physical activity in children. Further studies are required to determine whether an exercise program exceeding two years, with a higher intensity of training, in girls with a lower everyday level of physical activity and in early peri-pubertal girls could be beneficial for the FN structure.

## Competing interests

The authors declare that they have no competing interests.

## Authors' contributions

GA and HA were involved in the statistical analysis, the interpretation of data and the writing of the manuscript; CL, JB and SSL collected all data except the accelerometer data and were involved in the drafting of the manuscript; MD collected and analyzed the accelerometer data; PG designed the study and was involved in collecting the data; MK designed the study, worked with the analysis and the interpretation of data and was in charge of writing the manuscript. All authors read and approved the final manuscript.

## References

[B1] Kannus P, Haapasalo H, Sankelo M, Sievanen H, Pasanen M, Heinonen A, Oja P, Vuori I (1995). Effect of starting age of physical activity on bone mass in the dominant arm of tennis and squash players. Ann Intern Med.

[B2] Bass S, Pearce G, Bradney M, Hendrich E, Delmas PD, Harding A, Seeman E (1998). Exercise before puberty may confer residual benefits in bone density in adulthood: studies in active prepubertal and retired female gymnasts. J Bone Miner Res.

[B3] Bailey DA, McKay HA, Mirwald RL, Crocker PR, Faulkner RA (1999). A six-year longitudinal study of the relationship of physical activity to bone mineral accrual in growing children: the university of Saskatchewan bone mineral accrual study. J Bone Miner Res.

[B4] Forwood MR, Baxter-Jones AD, Beck TJ, Mirwald RL, Howard A, Bailey DA (2006). Physical activity and strength of the femoral neck during the adolescent growth spurt: a longitudinal analysis. Bone.

[B5] Ahlborg HG, Johnell O, Turner CH, Rannevik G, Karlsson MK (2003). Bone loss and bone size after menopause. N Engl J Med.

[B6] Haapasalo H, Sievanen H, Kannus P, Heinonen A, Oja P, Vuori I (1996). Dimensions and estimated mechanical characteristics of the humerus after long-term tennis loading. J Bone Miner Res.

[B7] Bass SL, Saxon L, Daly RM, Turner CH, Robling AG, Seeman E, Stuckey S (2002). The effect of mechanical loading on the size and shape of bone in pre-, peri-, and postpubertal girls: a study in tennis players. J Bone Miner Res.

[B8] Heinonen A, Sievanen H, Kannus P, Oja P, Pasanen M, Vuori I (2000). High-impact exercise and bones of growing girls: a 9-month controlled trial. Osteoporos Int.

[B9] McKay HA, Petit MA, Schutz RW, Prior JC, Barr SI, Khan KM (2000). Augmented trochanteric bone mineral density after modified physical education classes: a randomized school-based exercise intervention study in prepubescent and early pubescent children. J Pediatr.

[B10] MacKelvie KJ, Khan KM, Petit MA, Janssen PA, McKay HA (2003). A school-based exercise intervention elicits substantial bone health benefits: a 2-year randomized controlled trial in girls. Pediatrics.

[B11] Valdimarsson O, Linden C, Johnell O, Gardsell P, Karlsson MK (2006). Daily physical education in the school curriculum in prepubertal girls during 1 year is followed by an increase in bone mineral accrual and bone width--data from the prospective controlled Malmo pediatric osteoporosis prevention study. Calcif Tissue Int.

[B12] Linden C, Ahlborg HG, Besjakov J, Gardsell P, Karlsson MK (2006). A school curriculum-based exercise program increases bone mineral accrual and bone size in prepubertal girls: two-year data from the pediatric osteoporosis prevention (POP) study. J Bone Miner Res.

[B13] MacKelvie KJ, Petit MA, Khan KM, Beck TJ, McKay HA (2004). Bone mass and structure are enhanced following a 2-year randomized controlled trial of exercise in prepubertal boys. Bone.

[B14] Petit MA, McKay HA, MacKelvie KJ, Heinonen A, Khan KM, Beck TJ (2002). A randomized school-based jumping intervention confers site and maturity-specific benefits on bone structural properties in girls: a hip structural analysis study. J Bone Miner Res.

[B15] McKay HA, MacLean L, Petit M, MacKelvie-O'Brien K, Janssen P, Beck T, Khan KM (2005). "Bounce at the Bell": a novel program of short bouts of exercise improves proximal femur bone mass in early pubertal children. Br J Sports Med.

[B16] Linden C, Alwis G, Ahlborg H, Gardsell P, Valdimarsson O, Stenevi-Lundgren S, Besjakov J, Karlsson MK (2007). Exercise, bone mass and bone size in prepubertal boys: one-year data from the pediatric osteoporosis prevention study. Scand J Med Sci Sports.

[B17] Yoshikawa T, Turner CH, Peacock M, Slemenda CW, Weaver CM, Teegarden D, Markwardt P, Burr DB (1994). Geometric structure of the femoral neck measured using dual-energy x-ray absorptiometry. J Bone Miner Res.

[B18] Beck TJ, Oreskovic TL, Stone KL, Ruff CB, Ensrud K, Nevitt MC, Genant HK, Cummings SR (2001). Structural adaptation to changing skeletal load in the progression toward hip fragility: the study of osteoporotic fractures. J Bone Miner Res.

[B19] Khoo BC, Beck TJ, Qiao QH, Parakh P, Semanick L, Prince RL, Singer KP, Price RI (2005). In vivo short-term precision of hip structure analysis variables in comparison with bone mineral density using paired dual-energy X-ray absorptiometry scans from multi-center clinical trials. Bone.

[B20] Bradney M, Pearce G, Naughton G, Sullivan C, Bass S, Beck T, Carlson J, Seeman E (1998). Moderate exercise during growth in prepubertal boys: changes in bone mass, size, volumetric density, and bone strength: a controlled prospective study [see comments]. J Bone Miner Res.

[B21] Kohl III HW, Fulton JE, Casperson CJ (2000). Assessment of physical activity among children and adolescents: a review and synthesis.. Prev Med.

[B22] Sallis JF, Saelens BE (2000). Assessment of physical activity by self-report: status, limitations, and future directions. Res Q Exerc Sport.

[B23] Binkovitz LA, Henwood MJ (2007). Pediatric DXA: technique and interpretation. Pediatr Radiol.

[B24] Duke PM, Litt IF, Gross RT (1980). Adolescents' self-assessment of sexual maturation. Pediatrics.

[B25] Dencker M, Thorsson O, Karlsson MK, Linden C, Svensson J, Wollmer P, Andersen LB (2006). Daily physical activity in Swedish children aged 8-11 years. Scand J Med Sci Sports.

[B26] Dencker M, Thorsson O, Karlsson MK, Linden C, Svensson J, Wollmer P, Andersen LB (2006). Daily physical activity and its relation to aerobic fitness in children aged 8-11 years. Eur J Appl Physiol.

[B27] Dencker M, Thorsson O, Karlsson MK, Linden C, Eiberg S, Wollmer P, Andersen LB (2006). Daily physical activity related to body fat in children aged 8-11 years. J Pediatr.

[B28] Freedson PS, Sirard J, Debold E, Pate R, Dowda M, Trost S, Sallis J (1997). Calibration of the Computer Science and Applications, Inc (CSA) accelerometer. Med Sci Sports Exerc.

[B29] Trost SG, Ward DS, Moorehead SM, Watson PD, Riner W, Burke JR (1998). Validity of the computer science and applications (CSA) activity monitor in children. Med Sci Sports Exerc.

[B30] Mundy GR, Chen D, Oyajobi BO, Favus MJ (2003). Bone Remodeling. Primer on the Metabolic Bone Diseases and Disorders of Mineral Metabolism.

[B31] Janz KF, Burns TL, Levy SM, Torner JC, Willing MC, Beck TJ, Gilmore JM, Marshall TA (2004). Everyday activity predicts bone geometry in children: the iowa bone development study. Med Sci Sports Exerc.

[B32] Mackelvie KJ, McKay HA, Khan KM, Crocker PR (2001). A school-based exercise intervention augments bone mineral accrual in early pubertal girls. J Pediatr.

[B33] Morris FL, Naughton GA, Gibbs JL, Carlson JS, Wark JD (1997). Prospective ten-month exercise intervention in premenarcheal girls: positive effects on bone and lean mass. J Bone Miner Res.

[B34] Nikander R, Sievanen H, Heinonen A, Kannus P (2005). Femoral neck structure in adult female athletes subjected to different loading modalities. J Bone Miner Res.

[B35] Fuchs RK, Bauer JJ, Snow CM (2001). Jumping improves hip and lumbar spine bone mass in prepubescent children: a randomized controlled trial. J Bone Miner Res.

[B36] McKay H, Tsang G, Heinonen A, MacKelvie K, Sanderson D, Khan KM (2005). Ground reaction forces associated with an effective elementary school based jumping intervention. Br J Sports Med.

[B37] Kontulainen S, Sievanen H, Kannus P, Pasanen M, Vuori I (2003). Effect of long-term impact-loading on mass, size, and estimated strength of humerus and radius of female racquet-sports players: a peripheral quantitative computed tomography study between young and old starters and controls. J Bone Miner Res.

[B38] Brage S, Wedderkopp N, Franks PW, Andersen LB, Froberg K (2003). Reexamination of validity and reliability of the CSA monitor in walking and running. Med Sci Sports Exerc.

[B39] Rubin CT, Lanyon LE (1984). Regulation of bone formation by applied dynamic loads. J Bone Joint Surg Am.

[B40] Robling AG, Hinant FM, Burr DB, Turner CH (2002). Improved bone structure and strength after long-term mechanical loading is greatest if loading is separated into short bouts. J Bone Miner Res.

[B41] Saxon LK, Robling AG, Alam I, Turner CH (2005). Mechanosensitivity of the rat skeleton decreases after a long period of loading, but is improved with time off. Bone.

[B42] Robling AG, Hinant FM, Burr DB, Turner CH (2002). Shorter, more frequent mechanical loading sessions enhance bone mass. Med Sci Sports Exerc.

[B43] Turner CH, Robling AG (2005). Exercises for improving bone strength. Br J Sports Med.

[B44] Turner CH, Robling AG (2003). Designing exercise regimens to increase bone strength. Exerc Sport Sci Rev.

[B45] Heinonen A, McKay HA, Whittall KP, Forster BB, Khan KM (2001). Muscle cross-sectional area is associated with specific site of bone in prepubertal girls: a quantitative magnetic resonance imaging study. Bone.

